# Needs and Experiences of Children and Adolescents with Pediatric Multiple Sclerosis and Their Caregivers: A Systematic Review

**DOI:** 10.3390/children8060445

**Published:** 2021-05-25

**Authors:** Shashank Ghai, Elisabeth Kasilingam, Roberta Lanzillo, Masa Malenica, Vincent van Pesch, Niamh Caitlin Burke, Antonio Carotenuto, Rebecca Maguire

**Affiliations:** 1 School of Physical and Occupational Therapy, McGill University, Montréal, QC H3G 1Y5, Canada; 2 Feil & Oberfeld Research Centre of the Jewish Rehabilitation Hospital—CISSS Laval, a Research Site of the Centre for Interdisciplinary Research of Greater Montreal (CRIR), Laval, QC H7V 1R2, Canada; 3 Department of Family Medicine, McGill University, Montréal, QC GRM MDI, Canada; 4 European Multiple Sclerosis Platform, 1030 Brussels, Belgium; elisabeth.kasilingam@emsp.org; 5 Department of Neurosciences, Reproductive and Odontostomatological Sciences, Federico II University, 80138 Naples, Italy; robertalanzillo@libero.it (R.L.); carotenuto.antonio87@gmail.com (A.C.); 6 Department of Child Neurology, Associated Member of the ERN EpiCARE, Sestre Milosrdnice University Hospital Center, 10000 Zagreb, Croatia; malenicamasa96@gmail.com; 7 Department of Neurology, Cliniques Universitaires Saint-Luc, UCLouvain, 1200 Brussels, Belgium; vincent.vanpesch@uclouvain.be; 8 Department of Psychology, Maynooth University, Maynooth W23 F2K8, Ireland; niamh.burke.2019@mumail.ie (N.C.B.); rebecca.maguire@mu.ie (R.M.)

**Keywords:** pediatric multiple sclerosis, caregivers, burden, social support, quality of life

## Abstract

In the present study we conduct a systematic review to evaluate the needs and experience of people with pediatric multiple sclerosis (MS) and their caregivers. The literature search was conducted across 10 academic databases, adhering to PRISMA-P guidelines. Quality appraisal was conducted using the mixed method appraisal test for individual studies, and GRADE-CERQual to establish overall confidence of findings. Results were analyzed using a process of narrative synthesis. We identified 26 studies which included 2253 children/adolescents with MS (CAMS) and 1608 caregivers. MS was reported to negatively impact experiences for CAMS in domains such as of school performance, social relationships, mental health, and overall physical functioning. Specifically, fatigue and social support were reported as the most important barriers and facilitators for CAMS, respectively. In terms of caregiver experience, negative impacts were reported on social functioning, mental health, and quality of life. Additionally, lack of awareness concerning MS was one of the biggest challenges reported. Caregivers expressed needs for psychological and social support. This study provides the first evidence regarding the needs and experiences of CAMS and their caregivers. Findings can be used to address policy gaps for supporting families affected by pediatric MS.

## 1. Introduction

Multiple sclerosis (MS) is an autoimmune inflammatory and neurodegenerative disease characterized by demyelination of the central nervous system [1,2,3]. Until recently, MS was considered a disease that predominantly affects adults, but recent evidence has shown a growing prevalence of MS in pediatric populations (≤18 years old) [4,5]. A recent epidemiological study by Yan, et al. [6] reported an overall global prevalence of 8.1 (95% C.I: 2.2 to 13.9), and an overall global incidence of around 0.87 (0.3 to 1.4) out of 100,000 people. This rise in prevalence has been widely attributed to developments in diagnostic measures and increasing awareness among healthcare professionals [7,8,9].

Research evaluating the pathophysiology of pediatric MS has reported that as compared to adult onset MS, the disease course differs considerably in terms of progression [3,10,11]. For example, Fay, et al. [11] reported that pediatric MS cases experience higher and more severe relapse events when compared with adult-onset MS. Nonetheless, pediatric cases exhibit a rather more pronounced recovery than in adult cases [11], primarily because of their body’s enhanced ability to repair and/or lessen the extent of irreversible neural damage during relapses [3].

Children and adolescents with MS (CAMS) may exhibit a wide array of deficits in psychological [12], cognitive [13], sensory [14], and physical domains [15], which may be because disease onset coincides with the developmental stage of the central nervous system [16]. These manifestations may in turn impact negatively on various aspects of life in CAMS [3], including in the context of education [17]. Lack of awareness regarding the impact of MS among teachers and peers further adds towards the disease burden which may adversely impact quality of life (QOL) [18].

It is well established that caregivers (i.e., those who tend to the needs or concerns of a person with short- or long-term limitations due to illness, injury or disability [19]) of adults with MS can experience considerable burdens [20], and recent evidence suggests that this may also be true for caregivers of CAMS, who are predominantly parents [21,22]. These negative consequences may occur during different stages of the disease (i.e., pre diagnosis, diagnosis, and management) impacting on psychological, social, and employment-related domains [23,24]. As a result, caregivers of CAMS fare poorly in terms of health-related QOL outcomes when compared with parents of children suffering from monophasic acquired demyelinating disorder [25], and healthy children [26]. Feelings of guilt, uncertainty, and stress, coupled with concern for their child’s future, lack of proper knowledge about MS, and overloaded schedules have been suggested to be as factors which may aggravate detrimental psychosocial consequences [27,28,29].

Despite having a profound negative impact on CAMS and their caregivers, gaps in policy frameworks have been widely reported in terms of addressing the needs of these groups and providing evidence-based care [8,22]. These include gaps in provision of, and access to, adequate healthcare for CAMS, as well as the lack of a uniform policy to facilitate financial support for families caring for those with pediatric MS. This may be compounded by the considerable variation in healthcare systems internationally (e.g., whether there is a system of universal or single payer healthcare in place). One of the major reasons for policy gaps is the lack of systematically synthesized evidence reporting the needs and experiences of CAMS and their caregivers, thereby making it difficult for policy makers to take necessary steps to address these limitations. In this present systematic review, we attempt to address this gap by establishing a state of evidence regarding the needs and experience of CAMS and their caregivers. The findings from this review will serve to be a vital source of information for medical practitioners, educators, policy, and decision makers to take adequate steps to ensure the highest quality of support for CAMS and also for their caregivers.

## 2. Materials and Methods

A systematic review was carried out in adherence to Preferred Reporting Items for Systematic Reviews and Meta-Analyses guidelines. The review was preregistered at OSF registries (osf.io/9bfnx). A PRISMA-P checklist has been provided in the Supplementary File S1.

### 2.1. Data Search Strategy

We systematically searched 10 academic databases (Web of Science, PEDro, EBSCO, MEDLINE, Scopus, CENTRAL, EMBASE, PROQUEST, PsychInfo, Pubmed) from inception until September 2020. Appropriate search terms and inclusion/exclusion criteria were determined by an expert panel in the European Multiple Sclerosis Platform (EMSP) and are listed in the protocol. We also searched the reference lists of eligible studies and requested articles from personal libraries of the expert panel on EMSP for additional literature.

The inclusion criteria of the studies were categorized according to the SPIDER (Sample, Phenomenon of Interest, Design, Evaluation, Research type) approach [30]. We included studies evaluating: (1) Children and adolescent with MS and/or (2) Formal or informal caregivers of CAMS; (3) Needs and experiences of CAMS and their caregivers; (4) Existing services, supports and/or consultation models offered by healthcare, social and educational services for CAMS and their caregivers; and/or (4) Qualitative or quantitative studies (except case series, review articles); (5) Studies published in peer-reviewed academic journals or conference proceedings. Abstract screening was independently performed by two groups of authors (Group 1: S.G, Group 2: R.M, E.K, R.L, M.M, N.C.B, V.V.P, A.C) with full texts of articles screened by two authors (S.G. and R.M). In cases of ambiguity regarding the study eligibility, discussions were held in monthly group meetings between authors to seek a consensus. After the selection of the studies the following data was extracted from the eligible studies: author details, study aims, country of research, study design, descriptive information of caregiver and/or individual with CAMS (i.e., sample size, age, gender), time from diagnosis of CAMS, assessment tools utilized, and study outcome(s).

### 2.2. Quality Assessment

Methodological quality of the included studies was assessed using the Mixed Method Appraisal Tool (MMAT) [31]. The MMAT enables the appraisal of mixed methods designs, qualitative designs, and a variety of different quantitative design types (e.g., randomized controlled trials, nonrandomized designs and descriptive designs). This tool includes a distinct set of screening questions depending on the methodological approach employed which enables a score to be computed based on the number of criteria (0–100%) met for each study based on their design. Following the MMAT guidelines of Pace, et al. [31], the categorization of the studies was done as high (four criteria met), moderate (two to three criteria met), and low (less than one criterion met) quality. Overall, this tool gives an indication of the methodological quality of studies in isolation.

### 2.3. Data Synthesis

Results were analyzed using the process of narrative synthesis. Outcomes regarding needs and experiences of pediatric multiple sclerosis and their caregivers were classified according to their nature as per the GRADE-CERQual evaluation (Grading of Recommendations Assessment, Development, and Evaluation of Confidence in the Evidence from Reviews of Qualitative research) approach. This approach allows an amalgamation of review findings that can help in the development of recommendations used to support CAMS and their caregivers based on four main criteria: methodological limitations, coherence of the review findings, adequacy of the data, and the relevance of the studies to the review question. The GRADE-CERQual approach can be used to estimate the level of confidence i.e., high, moderate, low, or very low on the outcomes of a knowledge synthesis [32]. The assessment of GRADE-CERQual was derived from both the individual study quality appraisals (because of the MMAT screening), and a consideration of the extent to which the overall review findings address the objectives of the review.

Summary findings from the review were also shared with a consortium of 35 identified experts (hereafter termed the “expert group”), previously been established by EMSP. This group comprised of a variety of stakeholders with either professional experience in caring and supporting people with pediatric MS, or who had lived experience of MS as a young person or caregiver. Specifically, the expert group involved healthcare professionals, including pediatric neurologists, MS society representatives from various European countries, and patient and caregiver research partners at the EMSP. In order to gather feedback, a lay summary of the review findings was developed and emailed to this expert group in December 2020. Experts were given the opportunity to indicate if and how the experiences and needs of CAMS and their caregivers identified in the review resonated with their own experiences. A total of 10 responses were received from experts spread across Portugal, Spain, Romania, Greece, Germany, Denmark, Iceland, Belgium, and Italy.

## 3. Results

### 3.1. Characteristics of the Studies

**Figure 1:**
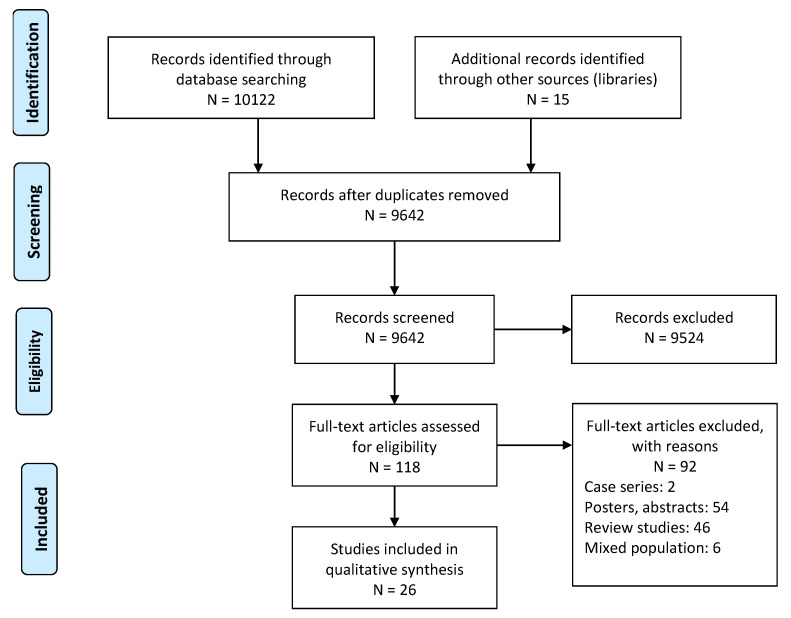
Illustrating the PRISMA flow chart for the included studies.

The initial search across 10 academic databases yielded a total of 10,122 studies (Web of Science: 197, PEDro:0, EBSCO:0, MEDLINE:8, Scopus:92, Cochrane central register of controlled trials: 6498, EMBASE:55, PROQUEST:80, PsychInfo:0, Pubmed:3192). We also included 15 additional studies from the personal libraries of the EMSP consortium. Thereafter, upon the implementation of our inclusion criteria the final number of studies was reduced to 26 (Figure 1). Of the 26 included studies, 11 were qualitative studies [18,23,27,28,33,34,35,36,37,38], 11 were cohort studies [24,25,26,39,40,41,42,43,44,45,46], and four were cross sectional studies [47,48,49,50]. Table 1 displays the summary characteristics of all studies included in the review.

#### 3.1.1. Country of Research

Of the included studies, eight were conducted in USA [23,29,34,35,36,43,48,49], and six were conducted in Canada [24,25,33,37,38,42]. Two studies were jointly carried out in Canada and USA [45,50]. Five studies were carried out in Italy [26,39,41,44,47], three in United Kingdom [18,27,28], one is Sweden [46], and one in Denmark [40].

#### 3.1.2. Population Characteristics

**Caregivers:** A total of 16 studies including a total of 1608 caregivers were included in our review [18,23,24,25,26,27,28,29,33,34,35,36,37,39,43,48,50]. Of the 16 studies, 15 reported that the included caregivers were parents of CAMS, one study however reported that although the majority of the caregivers were mothers (i.e., 19), for one study, an aunt was reported as the primary caregiver [34]. In the included studies, four did not specify the gender distribution of their caregivers sample [25,29,43,48]. The remaining reported data from 424 females and 129 males [18,23,24,26,27,28,33,34,35,36,37,39,50], whereas data from 653 females and 29 males were reported for the parents of healthy age matched children groups [24,26]. This demonstrates that mothers had a prominent role in caregiving. The average age of the caregivers was reported for six studies as (mean ± standard deviation): 42.2 ± 6.3 years for caregivers of CAMS [18,23,24,26,34,36], and 36.5 ± 9.8 years by two studies for the parents of healthy children [24,26].

**CAMS:** Data of 2253 CAMS were included in all 26 studies. Here, three studies did not report the gender distribution [29,34,47]. In the other studies which reported the gender distribution of their sample, data of 1378 females and 800 males was reported. The age was reported as range by three studies [29,37,39], and from the rest of the studies the calculated average age was reported as 16.2 ± 3.9 years. Average time to diagnosis of MS was reported by 19 studies [18,23,25,26,27,34,35,36,39,40,41,43,44,45,47,48,49,50], except one study which reported the values as range [33], the average time to diagnosis was reported as 2.8 ± 1.2 years. Further, the average Expanded Disability Status Scale scores of the CAMS was reported as 1.6 ± 0.4 by only 10 studies [36,38,39,41,43,44,45,47,48,49].

**Controls:** In addition to CAMS, some studies had also included a control group to comparatively evaluate the differences in the needs and experiences with that of CAMS. Here, twelve studies had included a comparative evaluation of CAMS with healthy children, children with other neurological disorders, and people with adult-onset multiple sclerosis [24,25,39,40,41,42,43,44,45,46,47,49]. In the healthy control group nine studies [24,40,41,42,43,45,46,48,49] reported the data of 9938 age matched controls. Here, a total of 6207 females and 3142 males were included in the healthy control group, with an average age of 14.7 ± 4.8 years, one study did not report the gender distribution and the average age of their healthy control sample [40].

Additional control comparison of pediatric other neurological disorders was reported by three studies [25,40,43]. It is important to note that two studies discussed here had also jointly presented data of healthy controls discussed in the previous paragraph [40,43]. Nonetheless, these three studies presented the data of 9324 children with neurological disorders. In the three studies, one did not report neither the gender distribution nor the age of their sample [40]. In the rest of the two studies [25,43], there was a total of 98 females, and 118 males, with an average age of 10.8 ± 2.5 years.

Besides this, two studies in our review also compared the outcomes between CAMS and people with adult-onset MS [44,47]. Here, data were reported for a total of 135 adults with MS. In these two studies, one study did not report the gender distribution and the age of their sample [47]. The other study by Portaccio, et al. [44], however, had reported the data of 68 females, 47 males with an average age of 27.3 ± 8.0 years.

### 3.2. Assessment

In the included studies different methods of assessments were used to assess the needs and experiences of CAMS and their caregivers. Here, while 10 of the included studies had used interviews to assess the needs of CAMS and their caregivers [18,23,27,28,29,33,34,35,37,38]. The other 16 studies had used heterogenous quantitative assessment tools to assess the impact of MS on quality of life of CAMS, overall medical visits, social impact, adherence to medications, school performance, cognitive performance, etc. A detailed list of the assessment tools for each study has been provided in Table 1.

### 3.3. Mixed Method Appraisal Tool

After a methodological appraisal of all the studies, 17 were reported to be of high quality [24,26,27,28,34,35,36,38,39,40,41,42,43,44,45,46,47,50], whereas nine were observed to be of moderate quality [18,23,25,26,29,33,37,48,49]. A detailed description of the methodological appraisal for the quantitative nonrandomized trials and qualitative studies has been provided in Table 1. The majority of bias in cohort and cross-sectional studies were found because of lack of clarity regarding the presence of confounders in the study’s design and incomplete data outcomes, respectively. In qualitative studies, the majority of bias was observed due to lack of coherence between different phases of the study, i.e., data collection, analysis, and interpretation. A detailed description of the included studies has been provided in Table 1.

### 3.4. Synthesis of Findings and Grade-CERQual Assessment

The findings of the included studies were thematically analyzed according to the reported experiences of both CAMS (Table 2) and their caregivers (Table 3). This analysis illustrates the negative impacts that pediatric MS has on various domains of CAMS and caregiver functioning, as well as highlighting a number of barriers and facilitators to more positive outcomes in both these groups. In relation to caregivers, we also note evidence in relation to experiences at diagnosis and future concerns. This information is also presented in the context of the Grade-CERQual criteria suggesting some variability in confidence of these experiences.

Drawing on this information, an assessment of needs for families of CAMS was also developed (Table 4). This suggests a high level in confidence in existing needs for psycho-logical and social support, as well as additional information in relation to MS for families.

## 4. Discussion

Gaps in the existing literature with respect to the understanding of the incidence and outcomes of pediatric PMMS have been widely documented. International support groups including the World Health Organization [51] and Multiple Sclerosis International Federation [8], have repeatedly called upon the scientific and healthcare community to develop a state of evidence that clearly delineates the impact of pediatric multiple sclerosis on both children/adolescents and their caregivers. This present review addresses this gap by establishing a state of evidence regarding the needs and experiences of CAMS and their caregivers. We discuss the findings with respect to the experiences of CAMS and caregivers during and after the diagnostic phase of the disease and its impact on their activities of daily living.

### 4.1. Diagnostic Phase

One finding from the review was the difficulties associated with the diagnostic process itself, particularly from the point of view of the caregiver. An MS diagnosis may be delayed and difficult due to a lack of understanding among healthcare professionals and difficulties distinguishing diagnoses with that of associated demyelinating disorders (e.g., acute disseminating encephalomyelitis) [9]. Hinton and Kirk [27] after interviews with parents of CAMS, reported that misinterpretation of the symptoms of disease by the primary care practitioners was common. Medical practitioners lacked knowledge in interpreting neuroimaging reports, and sometimes confused MS incidence with other psychosocial developmental issues, resulting in delayed referral and diagnosis. Hebert, et al. [35] identified similar discrepancies in terms of diagnosis, reporting that an average of 3.6 ± 2.0 clinical visits were needed in a cohort of 42 children before receiving a diagnosis. This could possibly increase the financial burden on the family [52], and lead to a delay in the implementation of preventative therapies, eventually worsening the prognostic outcome of MS [3,35,53,54]. We identified the pre-diagnosis phase to be associated with aggravated levels of emotional distress (moderate confidence) especially for caregivers. One clear way to reduce such distress may therefore be to increase awareness of pediatric MS not only in neurologists and radiologists, but also pediatricians, pediatric neuropsychiatrists, and general physicians involved in the primary health care system. In addition to this, financial support, increased accessibility to a range of treatments, and coordinating referrals for psychological support to families during the pre- and postdiagnosis phases, may further alleviate emotional distress.

In our data synthesis, we identify, with a high level of confidence, that additional information about the course of the disease is needed by caregivers. We found that the process of communicating diagnosis to families served as another source of emotional distress for caregivers [27,29,35]. Some complained about receiving an overwhelming amount of information which left them feeling confused. While we might expect that specialist pediatric MS neurologists or those working in specialist MS centers should be better placed to disseminate information appropriately, increasing awareness and understanding of MS among all clinicians working with CAMS could enable them to disclose information in a manner that may reduce emotional distress associated with disease uncertainty in families. Moreover, our review found that, because diagnosis can be a shocking experience for parents and families of CAMS themselves, they might not be equipped to deliver this information to their children. This may be mitigated by providing advice to families regarding how to convey the diagnosis information to their children or by conveying diagnosis information in a tailored manner (e.g., providing tailored material about the prospective impact of MS with a multidisciplinary team including pediatric neurologists, psychologists to mitigate experiences of anxiety in both CAMS and caregivers) An additional aspect that may reduce emotional distress in CAMS is encouraging their own involvement during the diagnosis and decision-making process. This may allow them to develop a self-perception of control over MS and foster better treatment adherence [35,55].

### 4.2. Physical & Psychological Impact of MS

The multifaceted impact of MS on both the physical and psychological health of CAMS is now recognized. In the physical domain, fatigue is one of the major challenges faced by CAMS in carrying out basic activities of daily living [18,41,48,49,56]. Our synthesis found that physical fatigue acted as a deterrent for many activities, especially in coping with school related activities. Amato, et al. [39] reported a high prevalence of fatigue related outcomes in almost 73% of their cohort with MS, with fatigue-related symptoms associated with increased absence from school and cocurricular activities. Likewise, Carroll, et al. [18] reported that fatigue had a pronounced negative influence on school-related activities. CAMS in this study likened fatigue to a feeling of “wearing a giant sandbag” which eventually reduced their ability to carry out activities of daily living. Our review also identified caregivers to perceive the negative influence of fatigue on school performance. Yeh, et al. [45] for instance, reported that parents of CAMS perceived the school related performance of their child to be poorer as compared to parents of age-matched healthy controls. Here, mismanagement of time because of fatigue-related symptoms, the negative impact of fatigue on mental health, and lack of knowledge regarding how to manage fatigue-related symptoms were reported by caregivers as some factors leading to poorer school-related performance in CAMS [18,39,57,58]. Moreover, to add towards the burden of MS, a lack of awareness among teachers was reported in some of the studies reviewed [18,29,33,39]. Here, one study reported that teachers commonly misinterpreted fatigue-related symptoms of children as “laziness” [18,59]. As a consequence, increased pressure of school-related work and poorer academic performance could predispose CAMS towards poorer mental health and possibly negatively influence employment-related outcomes later on [40]. In our synthesis, we found with high overall confidence that fatigue is a prominent barrier for CAMS and also that school-related performance is negatively impacted. While there clearly are other factors which may impact on the experience of school and cognitive function in this population that were not explored in this study (e.g., lesion burden and location), these findings suggest that identifying ways to mitigate fatigue may be one way of facilitating a more positive educational experience for CAMS. Similarly, we have moderate confidence that lack of teacher’s knowledge acts as a barrier for CAMS, with teachers’ misinterpretation of symptoms common. This may be due to poor levels of awareness of PMS in the current educational system [18], and families’ miscommunication of the disease diagnosis to teachers, due to fear of prejudice and/or embarrassment [23,29,33]. Increased educational support, by means of both increased awareness and disease specific accommodations for CAMS by the school could help resolve this challenge [29]. We also recommend the facilitation of frequent lines of communication between parents and teachers to discuss disease-related outcomes. Previous studies have documented that disclosures of other pediatric diseases in the educational context led to enhanced accommodations in the teaching environment allowing children to better cope with the disease [27,60,61].

### 4.3. Social Impact of MS

Another important consequence of MS which is often overlooked is the social impact of the disease on both the child and their caregiver [12]. In CAMS, fatigue-related reduction in physical activity was identified as one of the major aspects that led to the worsening of peer relationships. Carroll, et al. [18], for instance, reported that the inability of CAMS to cope with peers forced them to opt-out of social relationships because of feelings of guilt. Failing to disclose diagnosis to peers because of fear of repercussions (e.g., differential treatment) was considered an additional aspect that negatively impacted social relationships of CAMS. An important area to look into is disease-related stigma prevention in the educational settings by peers and educators. Besides, we found with high confidence that providing additional social support to CAMS could be important in improving their quality of life. As fatigue and other unpredictable symptoms, such as relapses, as well as complexities in social relationships with peers and difficulties in educational settings result in experiences of mental health issues for CAMS (high level confidence finding), adequate psychological support should be provided to CAMS. In collaboration with patient organizations, adequate responses to this unmet need should be established to ensure that psychological support that suits CAMS according to their disease course stage, such as in-patient organizations groups or in educational and medical settings [21,62].

Similarly, caregivers reported a negative impact of MS on their social lives. Hinton and Kirk [28] reported that parents of CAMS suffered in terms of their social interactions primarily because of time constraints due to disease management and increased instances of disputations with peers, especially while discussing the diagnosis of their child. In addition to worsened peer relationships, both CAMS and their parents reported negative influences of the disease burden on familial relationships. Cross, et al. [23] reported that MS exerted extra demands on a family in terms of disease management. Moreover, the authors mentioned that the extra demands on the family resulted in strained marital and sibling relationships. Harris [34] too reported poor familial relationship outcomes especially between siblings, citing poor communication between family relations regarding adjustment and adaptation as the main reasons for this decline. In our review, we found that MS had an overall negative influence on social outcomes in both CAMS and their caregivers with a high level of confidence. In order to mediate this negative impact on social outcomes, a need for increased social support for both CAMS and their caregivers was identified in our data synthesis. Here, the organization of community support events by MS communities, to bring existing families of CAMS in contact with each other, was considered as a helpful measure to cope. Moreover, the organization of summer camps in which CAMS discuss their day-to-day experiences was reported to promote coping strategies [29,63].

### 4.4. Limitations

While this review provides good evidence of the various physical, psychological and social impacts that can be experienced by CAMS and their caregivers, there are likely to be many other factors that impact on these experiences that were not captured in the review. For example, socioeconomic factors, such as the cost of treatment, availability of health insurance, and access to appropriate healthcare, are likely to exacerbate difficulties for families impacted by pediatric MS. While we found no evidence to suggest any differences in experience of those living in countries differing in the whether or not there was provision of a universal healthcare system, it seems reasonable to expect that those unable to access healthcare would be more negatively impacted. Furthermore, our review did not capture differences in experiences according to type of MS, extent of progression or imaging severity. Since most of the studies included in the review comprised of participants with a low EDSS (e.g., 10 studies included CAMS with an EDSS of less than 1.6), the needs and experiences of CAMS with higher levels of disability and their caregivers may not be reflected in the review findings. Finally, there may be some limitations relating to the methodologies used to capture experiences in the included studies. For instance, the absence of quantitative measurements for some variables means that these can only be interpreted subjectively.

## 5. Conclusions

This systematic review presents the first detailed synthesis of current evidence regarding the needs and experiences of CAMS and their caregivers. Most importantly, the findings of the present review resonated with the experiences of MS patient organizations, expert members, MS support groups, and patient research partners affiliated with the European Multiple Sclerosis Platform. We identify specific gaps in the existing policies and support systems based on the experiences of CAMS and their caregivers. We also identify specific needs in terms of psychological, social, educational, informational, and financial support which the policymakers and existing support systems can specifically use to bridge the gaps in existing policies and enhance the quality of care to both CAMS and their caregivers.

## Figures and Tables

**Table 1:** Illustrates the Grade-CERQual criteria from the included studies for the experiences of CAMS.

Authors	Country	Design	Descriptive CAMS	CAMS Age (Years)	Time from Diagnosis (Years)	Descriptive Caregiver	Caregiver Age (Years)	Assessments	MMAT Quality	Outcome
McKay, et al. [46]	Sweden	Cohort study	CAMS: 485 (348F, 137M) Ct: 4850 (3480F, 1370M)	CAMS: 32 (26–40) Ct: 32 (26–40)	-	-	-	Education, coefficient of annual earning, disability benefits	High	Significantly lower frequency of CAMS (40.8%%) achieved postgraduate education than Ct (45.9%). Lower coefficient of annual earnings for CAMS as compared to Ct. Higher number of sick abseence and diability pension for CAMS as compared to Ct.
Boesen, et al. [40]	Denmark	Cohort study	CAMS: 92 (68F, 24M) Ct: 920 (-) CW-NBD: 9108 (-)	CAMS: 22 (16.2–27.3) Ct: - CW-NBD: -	6.1 (7–9.4)	-	-	School performance (secondary/high school), psychiatric comorbidity recognized by registration at (registration at relevant healthcare centers/services), and healthcare visits (primary care centers, hospitals visits and hospital admissions)	High	No difference for school performance in CAMS, CW-NBD, and Ct. Higher rate of psychiatric comorbidity in CAMS compared to Ct and CW-NBD. Higher hazard rates for CAMS for psychopharmacological drug redemptions and out-of-hospital psychiatrist/psychologist visits compared to CW-NBD. Higher healthcare utilization in CAMS than Ct during follow-ups (30-day, 1-year, 5-year), and for primary care centers, hospitals visits and hospital admissions. Higher healthcare utilization in CAMS than CW-NBD during follow-ups (30-day, 1-year, 5-year), for hospitals visits and hospital admissions, but not for primary care centers.
Portaccio, et al. [44]	Italy	Cohort study	CAMS: 111 (74F, 37M) AOMS: 115 (68F, 47M)	CAMS: 15.5 ± 2.3 AOMS: 27.3 ± 8.0	CAMS: 1.4 AOMS: 11.5	-	-	Social status (BSMSS), work and social adjustment (WSAS), occupational complexity (Class complexity 1–4), unemployment rate, cognition (BRB test, Stroop test, NART), depression (MADRS), fatigue (FSS), and IQ	High	Lower frequency of CAMS (12%) achieved postgraduate education than AOMS (19%). Significant impact of disability on employment, dependent upon extent of disability. Higher prevalence of CAMS (13%), as compared to AOMS (5%) achieving lower educational status as compared to their parents. CAMS patients exhibited lower educational levels had lower IQ as compared to CAMS patients with higher educational levels. Cognitive impairment observed (34.5%) without differences between CAMS & AOMS.
Marrie, et al. [24]	Canada	Cohort study	CAMS: 222 (94F, 128M) Ct: 616 (376F, 240 M)	CAMS: 13.1 ± 5.0 Ct: 13.1 ± 5.0	-	Mothers of CAMS: 156F Mothers of Ct: 624F	Mothers of CAMS: 29.7 ± 5.0 Mother of Ct: 29.6 ± 4.8	Number of medical visits and prevalence of physical and mental (anxiety, mood disorder) conditions prediagnosis, diagnosis and postdiagnosis (since ±5 years of diagnosis)	High	Higher prevalence of physical conditions, mood and anxiety disorder in mothers of CAMS than Ct during pre, during and post diagnosis of their child. Higher odds of psychiatric visit for mothers of CAMS. No differences in primary care visits between mothers of CAMS or Ct.
Yeh, et al. [38]	Canada	Qualitative study	28 (20F, 8M)	16.01 ± 1.84	-	-	-	Motivational interview regarding barriers and facilitators associated with adherence to disease modifying MS therapy	High	Adherence to medication dependent on creating and maintaining healthy habits. **Barriers to adherence included:** **Forgetting** due to disruption in routine as a result of demands from school, spending time with friends, doing extracurricular activities and travelling. Onset of fatigue reported as another prominent barrier. **Emotional insecurity**, including a fear of being judged, being treated differently or being embarrassed. **Experience with medications**, such as, negative emotional (e.g., nervousness) or physiological (e.g., pain) responses with respect to administration of medicine. **Facilitators of adherence included:** **Remembering** by using cues such as location of, maintaining a schedule, keeping up reminders, and using organizational tools like alarms and notebooks. **Intrinsic motivation**: Being able to manage symptoms by preventing future relapses, improvement of past symptoms, management of present symptoms, keeping health as top priority and having a desire to improve QOL **Extrinsic motivation**: Obeying authority (e.g., healthcare professionals), and advice especially from parents **Emotional security:** Receiving and providing emotional support created safe environment to build and maintain healthy medication habits. Developing awareness among peers and gaining respect of others was helpful in developing a safe space, especially for disclosing CAMS diagnosis. **Experience with medications:** Implementing coping strategies such as, distractions, self-motivating statements and making comparisons with worst case scenarios were sub-factors which served as a facilitator for increased adherence with medications.
Marrie, et al. [42]	Canada	Cohort study	CAMS: 659 (410F, 249M) Ct: 3294 (2050F, 1244M)	CAMS: 14.1 ± 4.5 Ct: 14.1 ± 4.5	-	-	-	Hospitalization rate and number of physician visits	High	Higher odds of hospitalization and ambulatory physician visits in CAMS than Ct. Higher CAMS visits to primary care (twofold), psychiatry (threefold), ophthalmology (18-fold) and neurology (100-fold) than Ct.
Cross, et al. [23]	USA	Qualitative study	21 (15F, 6M)	14.7	1.6	Parents: 21 (19F, 2M)	43.8	Interviews regarding symptoms prediagnosis, receiving diagnosis, adapting to life, treatment, family life, school, and living with CAMS over time	Moderate	The main themes to emerge were: **Prediagnosis**: Considerable stress after the onset of symptoms due to uncertainty and anxiety over possible diagnosis. **Receiving the diagnosis:** The presence of health care experts was helpful as they explained treatments options and prognostic outcomes. Reports regarding the information to be overwhelming was also documented. **Reaction to diagnosis**: Reactions included feelings of “shock”, “desperation”, “sadness”, and “fear”. Different approaches regarding disclosing diagnosis (ranging from maintaining privacy to being completely open with others). **Emotional impact**: Constant anxiety regarding symptoms, relapse and progression. Feelings of depression and being culpable as they felt they contributed in the development of MS through hereditary means. **Treatment**: Feelings of uncertainty with variations in the role of the CAMS in the decision-making process reported. Subcutaneous and intramuscular injections reported to cause anxiety or disgust in CAMS and families. Stress related to MRI associated with fear of discovery of lesions. **Impact at school**: Cognitive and physical symptoms caused impairments associated with learning and normal functioning. Communication regarding CAMS was a major issue and feelings of “embarrassment” reported. **Family life**: Extra demands due to CAMS in terms of organizing transportation, diagnostics, medications, and communicating with healthcare facilities. Negative impacts on employment, martial relationships, and sibling relationships. Positive effects also reported such as added value to family relationships after diagnosis. **Multiple sclerosis community**: Supports i.e., organized events, financial support, informational support by NMSS were beneficial. **Concerns for future**: Concerns regarding the outcome of MS in future, especially the fear of parents not being able to take care of their child, and effectiveness, costs of medications in future.
Hebert, et al. [35]	USA	Qualitative study	41 (32F, 9M)	17.6 ± 4.7	4.2 ± 3.2	Parents: 42 (40F, 2M)	-	Semistructured interviews regarding children and familial experience, during diagnosis, the impact of MS on family, educational and social life, and the impact on parents hopes and concerns for future of child	High	The main themes were: **Diagnosis:** Large number of discrepancies in terms of diagnosis i.e., lack of knowledge or negligence reported on the behalf of physician (clinical visit before diagnosis: 3.6 ± 2.0). **Parental reaction**: Parents reported being “scared” and “overwhelmed”, followed by feeling “shocked”, and a “sense of relief” after initial diagnosis, attributed to lack of awareness regarding MS.
O’Mahony, et al. [25]	Canada	Cohort study	CAMS: 58 (39F, 19M) monoADS: 178 (81F, 97M)	CAMS: 17 monoADS: 12.6	CAMS: 3.1 monoADS: 3.5	Parent of CAMS: 49, Parents of child with monoADS: 149	-	Pediatric quality of life reported by child and parents, multidimensional fatigue scale (child self-report, parent report of child’s health), and family impact module	Moderate	Poorer HrQOL for parents and CAMS in all the family impact module dimensions (compared to monoADS). No difference in HrQOL, physical and psychological wellbeing between CAMS and monoADS. Parents of CAMS reported poorer HrQOL (overall family impact score, parental emotional functioning, parental communication, parental worry, family functioning summary score, family relationships) in cases where second clinical episode (i.e., with good clinical outcome) was absent as compared to monoADS cases with full recoveries.
Schwartz, et al. [50]	Canada, USA	Cross sectional study	66 (44F, 22M)	15.4 ± 2.02	2.2 ± 2.2	Parents: 132 (66F, 66M)	-	Pediatric quality of life inventory, patient reported multiple sclerosis self-efficacy scale, and multiple sclerosis treatment adherence scale	High	Poorer patient related physical functioning correlated with lower medication adherence. Better self-reported parental physical functioning correlated with enhanced medication adherence. Parents were associated with higher levels adherence of medications in CAMS with poorer pediatric QOL, school functioning, and lower multiple sclerosis self-efficacy control. Oral disease modifying therapies associated with lesser parental involvement with adherence.
Harris [34]	USA	Qualitative study	20	17.3 ± 3.4	3.8	Mother: 19F Aunt: 1F	44.35 ± 6.60	Interviews with caregivers of CAMS regarding their experiences with CAMS prior to diagnosis, during the diagnosis and after the diagnosis	High	Caregivers reported stressors which arose before the diagnosis of CAMS affected their perceptions of demands for caregiving. Preconceived thoughts regarding CAMS affected initial reactions to diagnosis. Those identifying resources e.g., community or spirituality for coping better adapted to diagnosis. Lack of knowledge regarding CAMS, and unstable disease progression associated with poorer adjustment. Moreover, the additional needs of CAMS influenced the adjustment and adaption of caregivers. Changes in family role due to diagnosis and additional responsibilities was reported to have a negative impact on siblings of CAMS. A lack of communication was reported in between families of CAMS regarding adjustment and adaptation approaches in CAMS. A substantial impact of the child’s health status was reported on balancing ADL.
Hinton and Kirk [28]	UK	Qualitative study	23 (15F, 8M)	15	-	Parents: 31 (20F, 11M)	-	Semistructured interviews with parents to evaluate experiences during prediagnosis, diagnosis and postdiagnosis period.	High	Parents’ experiences associated with a feeling of “living with uncertainty”. **Diagnostic uncertainty**: Diagnosis process was lengthy and frightening, due to rarity of the condition and interpretation of fluctuating symptoms. Conflicts in medical opinion and a lack of definite diagnosis increased uncertainty. **Daily uncertainty**: Daily events defined as “unpredictable” and “uncertain”. Inability to predict outcome of disease made it difficult to manage illness along employment, familial responsibilities, social activities and normal family events. Lack of access to reliable information and professional support contributed to uncertainty. **Interaction uncertainty**: Disclosing child’s symptoms of MS in social interactions met with disputations. Healthy appearance of CAMS made it difficult to convince social groups as well as healthcare practitioners of the medical condition. **Future uncertainty**: Lack of knowledge by medical specialists created uncertainty regarding child’s future. Fear of future impairment and needs for extensive support bothered parents with worries regarding their future role. **Management strategies**: **Information search**: Searching of information (i.e., from medical practitioners, friends, family, internet, charities) post-diagnoses alleviated uncertainty. However, initial optimism was replaced by frustration when it was evident that medical specialist knowledge was limited and that the information available for undesirable and that the “anticipated sense of control was not realized”. Uncovering negative stories while searching the information increased uncertainty. **Continuous monitoring**: Observing, monitoring and documenting changes in child’s behavior, physical activity was also mentioned as a measure to reduce uncertainty. This was reported to allow a greater sense of control over their child’s health. Concerns regarding misinterpretation of signs increased uncertainty. **Implementing changes**: Modifying diet (e.g., nutritious food, Vitamin D), reduced uncertainty regarding potential relapses. However, uncertainty regarding the lasting effects of the diet on the child’s illness increased the parent’s uncertainty regarding the extent to which they can implement these changes. **Optimistic thinking**: Having an optimistic outlook regarding illness reduced uncertainty. The variability of symptoms and professional disputes between medical practitioners aided optimistic outcomes that their child might have been incorrectly diagnosed. Increased optimism was also aided by focusing more on the immediate present rather than the future. Some withdrew from potential sources of supports i.e., peer support groups citing they did not want to be reminded of MS.
Yeh, et al. [45]	Canada, USA	Cohort study	CAMS: 25 (14F, 11M) Ct: 27 (16F, 11M)	CAMS: 16.3 ± 1.8 Ct: 15.7 ± 2.5	CAMS: 2.21 Ct: 2.58	-	-	Adherence information from pharmacy fills, Morisky adherence, MSTAQ, parental involvement in DMT administration, PROM reflecting WOL cognitive functioning (MS neuropsychological screening assessment questionnaire), MSSE, Ryff scale of psychological well-being scale, and PDDS (Patient reported outcome of disability)	High	Reduced adherence using MEMS cap reports after three and six months’ follow-up for CAMS compared to Ct. Increased pharmacy refills reported after 6-months in CAMS compared to Ct. Reduced PEDS school function in parents of CAMS compared to parents of Ct. Enhanced Morisky adherence, self-efficacy, patient reported QOL, MSSE function and control scales in CAMS compared to Ct. Reduced MSTAQ barriers, PEDS school function, Ryff (self-acceptance, environmental mastery) well-being in CAMS as compared to Ct.
Carroll, et al. [18]	UK	Qualitative study	15 (8F, 7M)	15.2	2.9	Parents: 13 (11F, 2M)	46.8	Semistructured interviews to evaluate the effects of fatigue on experiences of parents and their CAMS	Moderate	The themes were: **Lived experience of fatigue and impact on ADL**: **CAMS**: Feelings of physical fatigue defined as “like wearing a giant sandbag” and cognitive fatigue as “like looking through a haze”. Interference in school, social and family life reported. Specifically, disruption in memory, concentration and attention affected activities in school. Poor sleep quality also reported that gave rise to a feeling of being “wiped out”. **Parents**: Arranging schedule according to CAMS’s fatigue was reported to affect ADL. **Uncontrollability and uncertainty of fatigue**: **CAMS:** Fatigue perceived as being uncontrollable. Some accepted fatigue as part of the disease and a manifestation that could not be changed. Moreover, uncertainty reported with respect to deciphering differences between normal childhood fatigue and fatigue associated with CAMS. **Parents**: Lack of available information exacerbated uncertainty and hindered ability to manage fatigue related symptoms. Feeling of “lack of control” reported. **Findings a balance**: **CAMS**: Difficulty in finding a balance between managing working and resting. **Parents**: Feeling of helplessness reported. Parents less inclined to encourage CAMS with activities as they wanted to give them freedom to manage MS related fatigue themselves. **Concern:** **CAMS**: - **Parents**: Concerns raised regarding implications of fatigue on mental health of CAMS. Concerns regarding future ability of CAMS to manage fatigue in adulthood when the load of responsibilities would be higher. **Social support and disclosure**: **CAMS**: Disclosing diagnosis was largely met with positive supportive responses from friends. Some participants did not disclose fearing differential treatment from peers, meaning that they got overexhausted to keep up with normal ADL’s with friends. Lack of knowledge regarding the diagnosis by teachers affected misinterpretation of fatigue as “laziness” and made normal schoolwork challenging. Feelings of “guilt” reported by CAMS as they felt culpable to limit their friends.
Hinton and Kirk [27]	UK	Qualitative study	21 (15F, 6M)	15 ± 2.36	1.91 ± 2.25	Parents: 23 (20F, 11M)	-	Semistructured interviews regarding the experiences of CAMS and their parents during diagnosis of MS	High	The main themes were: **Symptoms:** The majority developed gradual symptoms which prompted parents to seek medical advice. **Recognizing a problem:** CAMS reported to be reluctant to disclose symptoms. Some parents adopted a “wait and see” approach and tried to manage symptoms at home. **Seeking medical advice:** Continuation of symptoms or additional symptoms prompted parents to seek medical advice. Some parents reported that teacher remarks regarding the child’s health prompted them to seek medical advice. Most parents sought advice from a general practitioner at first. The influence of child’s willingness to seek medical advice was also a major factor in seeking this. **Communication concern:** While many (n=12) families reported being satisfied with the first healthcare consultation, others (n=11) reported being unsatisfied. Lack of tangible evidence regarding symptoms reported as one of the main concerns. Moreover, CAMS reported to feel that their concerns were disregarded without investigation. Parents were also suggested by medical practitioners to be imagining and overreacting to symptoms or even causing child’s health complaints. **Medical interpretation**: Frequent misinterpretation and delayed referral to secondary care frequently reported by parents. False attribution to viruses and psychosocial issues considered as the underlying reasons by healthcare practitioners. Lack of expertise to interpret MRI by pediatricians, and familiarity with CAMS reported in secondary care. Many (n=13) CAMS received an alternate diagnosis in secondary care prior to CAMS diagnosis. **Questioning medical opinion**: Increased frustration as a result of worsening of child’s symptoms and ignorance of medical practitioners led to feelings of “confusion” and “uncertainty” during the prediagnosis period. Parents were reported to take charge and seek secondary opinion ten consulted a different general practitioner, six requested further investigations, four presented at emergency care, and one payed for private consultation. Lack of medical knowledge or self-taught knowledge via internet search made it difficult to articulate parental concerns to medical practitioners. Uncertainty and struggles to communicate were a major cause of parental distress. **Receiving a CAMS diagnosis**: The time to diagnoses varied from one to 96 months after the initial onset of disease. Reluctance of pediatric neurologists to diagnose MS in childhood was reported to be a major factor for this delayed diagnosis. Moreover, uncertainty was widely reported regarding the accuracy of diagnosis due to conflicts in medical opinion, this uncertainty was also linked with “hope” for the parents that thought their child’s condition might improve. Medical practitioners which conveyed information in simple comprehensive terms while keeping in mind the emotional needs of the family were valued.
Lanzillo, et al. [47]	Italy	Cross sectional study	CAMS: 34 AOMS: 20	17.2 ± 3.6	3.5 ± 3.1	-	-	Peds QOL and brief repeatable battery of neuropsychological tests	High	HrQoL reported by Peds QOL higher in CAMS as compared to AOMS. No influence of cognitive impairment was reported on the quality of life for both CAMS and AOMS.
Krupp, et al. [29]	USA	Qualitative study	21	8–17	-	Parents: 30	-	Interviews	Moderate	**CAMS** Detrimental influences reported on physical activities and endurance. Negative impact on school performance and social relationships. Needs expressed with regards to being integrated in the consultancy phase of diagnosis. **Caregivers** Frustration reported at the time of diagnosis with respect to lack of knowledge and inability of medical professionals to make referrals. Some families reported sense of loss, while others reported a feeling of relief upon diagnosis. Negative impact on social relationships reported. Feelings of concern reported with respect to disease progression. Families supported the idea of including the CAMS during the phase of disclosure of diagnosis. Involvement with CAMS communities aided coping and adapting.
Thannhauser [37]	Canada	Qualitative study	9 (5F, 2M)	16–21	-	Parents: 6 (4F, 2M)	-	Semistructured interviews of experiences with PMS during the pre- and postdiagnosis phases.	Moderate	The main themes were: **Recurring loss**: Onset of psychological and emotional reactions discussed with respect to initial diagnosis. An avoidance behavior was reported by few CAMS during to convey the symptoms to their caregivers. **Suffering:** Period of diagnosis characterized with feelings of “shock”, “confusion”, “sadness”, “frustration”, depression, “hopelessness” and” anger”. **Fear of unknown**: The unpredictable nature of the illness led to fear with respect to future disabilities and loss of independence. **Losing trust:** The prediagnosis phase of testing considered as foreign, irrelevant and confusing. A distrust in the medical system was reported, in addition to feelings of “anger” and “frustration”. **Sense making**: Questions over making sense of themselves in the world following diagnosis were reported. CAMS were reported to use “sense making” to question their diagnosis and why they were diagnosed with it. **Carrying on**: All patients were reported to eventually develop a conscious choice regarding pursuing their future with MS. **Becoming me**: Transformative experiences were reported by CAMS. **Putting MS in its place**: Acceptance of MS as a part of their life was reported by CAMS. CAMS decided to prevent MS from taking control over their present/future as they tried to avoid stigma associated with the MS. **Pushing boundaries**: CAMS were reported to undergo mild to moderate risk-taking behaviors which allowed them to take control over their life. **Normalcy**: A concept of developing, maintaining and reinventing a sense of normalcy was reported. These feelings helped them to gain control and avoid the feelings of unpredictability associated with MS. It allowed them to shift their focus away from MS to gain normalcy in ADL. The CAMS also accepted the fact that due to their condition they might have to work harder as compared to others to achieve their goals. **Becoming expert**: A sense of becoming an expert in their life, their body and for their medical condition was reported. This expertise was demonstrated for five main aspects: controlling symptoms, making medical decisions, managing disease knowledge, advocating for self and planning the future. **Selective disclosing**: Disclosing the experiences of MS was associated with a feeling of “relief”. Selective disclosure of their diagnosis, symptoms was reported for to protect their personal self, reputation, and a means to cope with emotional experiences. **Judging readiness/openness to disclosure:** CAMS described a process of assessing the readiness of an individual i.e., emotionally to whom the disclosure was meant to be made. Feelings of “testing waters” were reported as they wanted to protect themselves from being emotionally vulnerable. **Developing cautious wisdom:** A shift in mindset was reported by CAMS for others’ perception of open mindedness towards a new sense of prejudice. **Meaning making**: All CAMS described ways they worked towards rebuilding their own narratives regarding the self and the world. **Perspective taking**: A more optimistic outcome was reported by CAMS for their uncertain future, especially by means of comparing themselves with people who were worse off. A sense of compassion was also reported towards dealing with others as a result of their own condition. **Reprioritizing**: Increased emphasis was reportedly placed by CAMS on family, close and intimate relationships after the diagnosis. A prioritization was also reported towards a healthy lifestyle (adequate sleep, limited alcohol, increased socializing). **Finding purpose**: A increased desire to find a purpose in their life which was adapted to their condition was also reported by CAMS. **Adopting an attitude of hope:** An attitude of finding a positive perspective among their struggles and barriers was reported by CAMS. Moreover, hope was another factor that came from sources e.g., new research, being an advocate for others with MS, and by living each day. **Turning points:** Multiple processes were reported which influenced the CAMS’s oscillation between recurring loss and carrying on in their life. **Labelling the disease:** Upon diagnosis a labelling of the disease allowed the CAMS to gain a sense of control a request for supports needed to manage ADL. **Shifting emotions**: Strange emotions were exhibited by CAMS upon diagnosis which they reported to be overwhelming. Moreover, the disease and the medications were reported to contribute towards development of depressive mood. However, CAMS reported that with passage of time they learned to manage their emotional experiences. **Managing medications:** Taking medications was a major factor disrupting daily routine of CAMS. Moreover, lack of feedback regarding medication’s effectiveness affected their adherence towards treatment. Using medications was “emotionally taxing” by both CAMS and their parents. The motivation for continuing medications for the individuals arose from their friends or significant others. **Dynamic relationships**: Positive impact of emotional support from medical practitioners, family and friends were reported on CAMS’s ability to process their loss and carry on. Reports of social relationships ending following MS diagnosis were also reported which contributed to the experience of loss.
Lulu, et al. [36]	USA	Qualitative study	30 (16F, 14M)	CAMS: 15.8 ± 2.8	2.5	Parents: 30 (23F, 7M)	46 ± 7.8	Peds QOL, IPQ, adherence questionnaire, TRAQ, HSAQ, SES, and SDMT	High	Nonadherence rate was reported in 37% of CAMS (30% for parents). Forgetting was the most common reason for nonadherence (50% for CAMS and 33% for parents). Higher EDSS score associated with lower Peds QOL score, and lower healthcare skill. Onset of relapse was associated with lower odds of adherence. Lower Peds QOL (psychosocial aspect) for parents as compared to CAMS. Benefit of using disease modifying therapy for CAMS was reported by their parents.
Uccelli, et al. [26]	Italy	Cohort study	CAMS: 14 (3F, 11M) Ct: -	CAMS: 13.7 ± 1.9 Ct: -	2.91 ± 3	Parents of CAMS: 30 (15F, 15M) Parents Ct: 58 (29F, 29M)	Parents of CAMS: 43.1 ± 3.5 Parents Ct: 43.5 ± 5.2	Maternal worry scale, ENRICH couple scale, WHO-five well-being index, HADS, PSOC, F-COPES, and multiple sclerosis knowledge questionnaire	Moderate	Higher depression score in HADS in parents with CAMS than Ct. Lower PSOC, and a need to seek spiritual support on F-COPES in parents with CAMS than Ct. Higher anxiety score for HADS in parents with CAMS as compared to normal parents. Lower WHO-five well-being index ENRICH score in parents with CAMS as compared to normal parents. **Gender differences between groups** Higher score for mothers of CAMS on the F-COPES seeking spiritual support than Ct. Higher depression score in HADS, the F-COPES seeking spiritual support for fathers of CAMS than Ct. Lower score in PSOC score, ENRICH conflict resolution subscale in fathers of CAMS as compared than Ct. **Gender differences within group** Significantly higher levels of ENRICH conflict resolution subscale, F-COPES total score in mothers as compared to fathers of CAMS. Higher score of multiple sclerosis knowledge questionnaire for mothers as compared to fathers of CAMS.
Goretti, et al. [41]	Italy	Cohort study	CAMS: 57 (31F, 26M) Ct:70 (37F, 33M)	CAMS: 16.6 ± 2.5 Ct: 16.0 ± 3.0	5.0 ± 3.5	-	-	Pediatric quality of life inventory—multidimensional fatigue scale, CDI, psychiatric interview through K-SADS-PL, parent reports of fatigue and cognitive deficits	High	21% of CAMS had depressive symptoms with CDI, and affective disorder with K-SADS-PL. Higher sleep and cognitive fatigue in CAMS than Ct. Higher general fatigue in CAMS than Ct. Higher sleep fatigue in CAMS than Ct as reported by parents. 9–14% of CAMS self-reports identified the presence of fatigue, whereas, 23–39% of parents of CAMS identified the onset of fatigue in their CAMS. Higher levels of fatigue correlated with higher scores of CDI. Higher levels of self-reported cognitive fatigue were associated with impaired problem-solving test performance. Higher levels of patient reported cognitive fatigue associated with impairments in tests of verbal learning, processing speed, complex attention and verbal comprehension.
Parrish, et al. [49]	USA	Cross sectional study	CAMS: 36 (25F, 11M) Ct: 92 (41F, 51M)	CAMS: 14.1 ± 3.6 Ct: 11.8 ± 3.7	2.1 ± 2	-	-	Self and parent reported depression evaluated by behavior assessment system for children second edition, Varni PedsQL Multidimensional fatigue scale total (sleep, cognitive, physical) fatigue	Moderate	**Parent reported**: Significantly higher levels of depressive score, total fatigue, i.e., sleep-related, cognition-related and general fatigue in CAMS. **CAMS reported**: Significantly higher levels of total fatigue, i.e., general and cognition related fatigue. Higher levels of self-reported depressive score and sleep-related fatigue.
Mowry, et al. [43]	USA	Cohort study	CAMS: 41 (31F, 10M) Ct with neurological disorder: 38 (17F, 21M) Ct normal: 12 (9F, 3M)	CAMS: 14 ± 4 Ct with neurological disorder: 9 ± 5 Ct normal: 13 ± 3	2.0	Parents of CAMS: 45 Parents of Ct with neurological disorder: 51 Parents of Ct normal: 10	-	Peds QOL (child self-report, parent proxy report)	High	Lower CAMS self-reported Peds QOL (total, school, social, psychosocial, physical) than healthy controls. Lower parent proxy Peds QOL for CAMS (total, school, emotional, psychosocial, physical) than healthy controls. Higher CAMS self-reported Peds QOL (physical and social) than Ct with neurological disorders. Lower parent proxy Peds QOL for CAMS (total, physical, psychosocial, and social) than Ct with neurological disorders.
MacAllister, et al. [48]	USA	Cross sectional study	51 (33F, 18M)	14.8 ± 2.2	1.6	Parents: 47	-	Child self-report, parent report of Peds QOL and Peds QOL multidimensional fatigue scale	Moderate	Self-reported fatigue correlated with sleeping difficulty, cognitive dysfunction, physical dysfunction, emotional dysfunction, and academic dysfunction. Parent reported fatigue correlated with sleep difficulty, cognitive dysfunction, physical dysfunction, emotional dysfunction, and academic difficulty. EDSS severity correlated with sleep difficulty, social dysfunction, and physical dysfunction. EDSS severity from parent-reports correlated with onset of fatigue, social dysfunction, cognitive dysfunction, physical dysfunction, and academic difficulties.
Amato, et al. [39]	Italy	Cohort study	CAMS: 63 (33F, 30M) Ct: 57 (32F, 25M)	CAMS: 15.3 ± 2.5 Ct: 14.8 ± 3.5	3.0 ± 3.2	Parents of CAMS: 41 (30F, 11M)	-	IQ, Expressive language, receptive language, neuropsychological test battery, parent interviews on child’s performance	High	**CAMS** Lower IQ (verbal and performance) in CAMS than in Ct. Cognitive impairment identified in 27% of cases where CAMS failed in three cognitive test domains, while 53% failed in two test domains. The most common impairment was in spatial recall. Cognitive impairment prevalence was 33% (age 8–13 years), 30% for other groups (14–18 years). Fatigue prominent in 73% of cases. Self-reported depressive symptoms in 6% of cases. **Parents**: Interviews revealed CAMS had significant impact on school activities and achievements. Only, 10% of CAMS had a support teacher. 22% S had to repeat school year because of missed days or cognitive dysfunction. Likewise, 34% reported a negative impact on hobbies and sports activities due to CAMS and 39% of CAMS had behavioral changes (anxiety, aggressiveness, isolation).
Boyd and MacMillan [33]	Canada	Qualitative study	12 (7F, 5M)	8–18	0.41–10	-	-	Interviews of experiences associated with diagnosis and coping with CAMS.	Moderate	The main themes were: **Learning the diagnosis**: Feelings of being “confused”, “scared”, “sadness”, and “pity” reported upon learning of diagnosis. Feelings of “relief” also reported by some CAMS as they finally had an explanation for symptoms. Knowledge of CAMS acquired via parents, healthcare professionals, printed information, self-experience, self-research via school projects, and internet. The most preferable means to obtain the information was described as to being able to read themselves or being talked by someone. **Noticing the difference**: Most important differences noted since CAMS diagnosis were: heat intolerance, followed by fatigue (5), headache (5), cognitive disabilities (5), sensory symptoms (4), hand tremor (2), seizures (2), depression (1), none (2). Experiences were reported with respect to not being able to perform fine and gross motor skills as before, having difficulties in carrying out school activities, being more cautious than before, and being treated differently than before. **Staying the same**: Continuing attending school, meeting friends and taking part in social activities was reported by all the CAMS even after diagnosis. Continuing personal activities of interests such as, reading, listening to music, playing on computer were also reported. **Coping with CAMS**: **Stressors**: A range of stressors were reported by CAMS that were related to treatment, symptoms, unpredictability of relapses, being treated differently, missing school, effect on family, restriction on lifestyle and uncertainty of future. **Strategies**: Maintaining a positive outlook on life, continuing to strive for their goals, and making downward comparisons with others having worse life conditions were some of the strategies CAMS implemented to cope. Remaining busy for distracting themselves from their condition, receiving support from others for dealing with CAMS. Negative influences as a mean to cope also were reported by some CAMS. **Gaining support**: Parents were defined by all CAMS as their main source of support. Moreover, the helpful role of friends and healthcare professionals was also mentioned as they encouraged them to accept their condition and provide information, respectively. **Dealing with treatment:** Discomfort associated with injection, side effects and cosmetic changes were defined as major aspects contributing towards stress. The injections were also defined as a source of regular reminder of their diagnosis. Some CAMS hid from their peer and family fearing a negative and judgmental outcome on their behalf. **Changing relationships**: Positive outcomes in relationship were reported between the family post-diagnosis. Teachers were reported maximally to misunderstand the needs of CAMS. **Peer response**: Most CAMS reported receiving a supportive response from their peers after disclosing the diagnosis. A few reported being downplayed by peers for their symptoms. Exclusions from activities from peers was also documented. **Disclosing diagnosis**: All the CAMS felt the need to disclose information to close family members, teachers, employers, and friends. Adolescents primarily felt others knowing the diagnosis as a matter of embarrassment and preferred to limit disclosure. **Effect learning**: Relapses and medical appointments were reported as a major factor for missing school. Moreover, cognitive deficits were reported to affect memory and concentration. Fatigue was also reported to be a main issue that affected their ability to finish a task. **Looking towards future**: All CAMS described having a feeling of “hope” about the future. None of the CAMS reported that they changed their career goals considering CAMS diagnosis.

AOMS: Adult onset multiple sclerosis. AMHS: Adult mental health services. ADL: Activities of daily living. BRB test: Brief repeatable battery of neuropsychological tests. BSMSS: Barratt simplified measure of social status score. CAMS: Pediatric multiple sclerosis. CAMHS: Children and adolescent mental health services. CDI: Children depression inventory. CW-NBD: Children with nonbrain related disorders. EDSS: Expanded disability status scale. F-COPES: Family crisis oriented personal evaluation scale. FSS: Fatigue severity scale. HCAQ: Health care assessment questionnaire. HADS: Hospital anxiety and depression scale. IQ: Intelligence quotient. IPQ: Brief illness perception questionnaire. JLOT: Benton judgement of line orientation test. K-SADS-PL: Kiddie-SADS-present lifetime version. MS: Multiple sclerosis. MMAT: Mixed method appraisal tool. MSSE: Multiple sclerosis self-efficacy scale. MSTAQ: Multiple sclerosis treatment adherence questionnaire. monoADS: monophasic acquired demyelinating syndrome. MADRS: Montgomery–Asberg depression rating scale. NART: National adult reading test. PSOC: Parenting sense of competence scale. PDDS: Patient determined disease steps. Peds QOL: Pediatric quality of life inventory. RSPM: Raven’s standard progressive matrices. SES: Socioeconomic status questionnaire. SDMT: Symbol digit modalities test. TRAQ: Transition readiness assessment questionnaire. WSAS: Work and social adjustment scale score. WHO: World health organization.

**Table 2:** Illustrates the Grade-CERQual criteria from the included studies for the experiences of CAMS.

	Experience of CAMS	Studies	CERQual Confidence	Explanation
**Negative impact on**	School performance	[18,23,25,27,29,33,37,38,39,40,43,44,45,47,48,50]	High	High relevance; minor coherence, data and methodology
	Social functioning	[18,23,25,29,33,34,35,37,38,43,44,45,48,50]	High	High relevance; minor coherence, data and methodology
	Mental health	[18,23,29,33,35,36,37,39,40,41,43,44,47,49]	High	High relevance; minor coherence, data and methodology
	Overall physical functioning	[25,26,29,34,37,39,40,42,43,47,50]	High	High relevance; minor coherence, data and methodology
	Quality of life	[25,36,43,45,47,48,50]	High	High relevance; minor coherence, data and methodology
	Later employment outcomes	[37,44,46]	Moderate	High relevance, moderate methodology, minor coherence and data
**Barriers**	Fatigue	[18,23,25,27,33,35,37,38,39,41,44,48,49]	High	High relevance; minor concerns about coherence, data and methodology
	Lack of teacher knowledge	[18,29,33,39]	Moderate	High relevance, moderate methodology, minor coherence and data
	Treatment adherence	[33,36,38]	Moderate	High relevance, moderate methodology, minor coherence and data
	Adverse effects of treatments	[33,42,50]	Moderate	High relevance, moderate methodology, minor coherence and data
**Facilitators**	Social support	[23,29,33,37,38,45,50]	High	High relevance; minor concerns about coherence, data and methodology
	Access to disease-modifying therapies	[23,36,43,45,50]	Moderate	High relevance, moderate methodology, minor coherence and data
	Provisioning of information and developing healthy habits	[28,29,37,38]	Moderate	High relevance, moderate methodology and data, minor coherence
	Motivation	[38,45]	Low	High relevance, moderate methodology, minor coherence and data

**Table 3:** Illustrates the information about the experiences of caregivers of CAMS.

	Experience of Caregivers of Pediatric Multiple Sclerosis	Studies	CERQual Confidence	Explanation
**Negative impact on**	Social functioning	[18,23,25,26,28,29,34]	High	High relevance; minor concerns about coherence, data and methodology
	Mental health	[23,26,29,34,42]	Moderate	High relevance, moderate methodology, minor coherence and data
	Quality of life	[25,26,43]	Moderate	High relevance, moderate methodology, minor coherence and data
	Employment	[26]	Very Low	High relevance, moderate methodology and data, minor coherence
**Diagnosis**	Emotional distress	[18,23,25,27,29,33,35,37]	High	High relevance, moderate methodology, minor coherence and data
	Healthcare professional’s presence helpful	[23,33]	Low	High relevance, moderate methodology, minor coherence and data
	Relief after receiving diagnosis	[29,35]	Low	High relevance, moderate methodology and data, minor coherence
**Future concerns**	Unpredictable outcome of the disease	[18,23,28,29]	Moderate	High relevance, moderate methodology, minor coherence and data
	Fear of unable to care the CAMS in future	[23,26]	Low	High relevance, moderate methodology, minor coherence and data
	Rising treatment costs	[26,41]	Low	High relevance, moderate methodology, minor coherence and data
**Barriers**	Lack of information regarding the disease	[18,23,28]	Moderate	High relevance, moderate methodology, minor coherence and data
	Lack of knowledgeable healthcare professional	[27,29,35]	Moderate	High relevance, moderate methodology, minor coherence and data
**Facilitators**	Provision of information on disease management	[18,23,25,29,34,35]	High	High relevance, moderate methodology, minor coherence and data
	Including CAMS in the decision-making process	[23,29,35]	Moderate	High relevance, moderate methodology, minor coherence and data
	Optimistic thinking	[28,33]	Low	High relevance, moderate methodology, minor coherence and data
	Intra-MS family communications	[23,29]	Low	High relevance, moderate methodology and data, minor coherence

**Table 4:** The needs of families of CAMS.

Needs of Caregivers of CAMS	Studies	CERQual Confidence	Explanation
Psychological support	[18,23,25,26,27,28,34,35,37]	High	High relevance; minor concerns about coherence, data and methodology
Social support	[18,23,26,28,34,35,37,43]	High	High relevance, moderate methodology, minor coherence and data
Additional information on disease	[18,23,26,27,28,34,35]	High	High relevance; minor concerns about coherence, data and methodology
Educational support	[18,23,34]	Moderate	High relevance, moderate methodology, minor coherence and data
Financial support	[23]	Very low	High relevance, moderate methodology and data, minor coherence

## Data Availability

Not applicable.

## Supplementary Materials

The following are available online at https://www.mdpi.com/article/10.3390/children8060445/s1, Supplementary File S1: PRISMA-P checklist.
